# Concurrent Overexpression of *OsGS1;1* and *OsGS2* Genes in Transgenic Rice (*Oryza sativa* L.): Impact on Tolerance to Abiotic Stresses

**DOI:** 10.3389/fpls.2018.00786

**Published:** 2018-06-21

**Authors:** Donald James, Bhabesh Borphukan, Dhirendra Fartyal, Babu Ram, Jitender Singh, Mrinalini Manna, Vijay Sheri, Varakumar Panditi, Renu Yadav, V. Mohan M. Achary, Mallireddy K. Reddy

**Affiliations:** ^1^Crop Improvement Group, International Centre for Genetic Engineering and Biotechnology, New Delhi, India; ^2^Department of Biotechnology, Uttarakhand Technical University, Dehradun, India; ^3^National Institute of Plant Genome Research, New Delhi, India

**Keywords:** glutamine synthetase, abiotic stress, herbicide tolerance, Glufosinate, *in vitro* gene pyramiding, Multi-Round Gateway technology

## Abstract

Glutamine synthetase (GS) is a key enzyme involved in the nitrogen metabolism of higher plants. Abiotic stresses have adverse effects on crop production and pose a serious threat to global food security. GS activity and expression is known to be significantly modulated by various abiotic stresses. However, very few transgenic overexpression studies of GS have studied its impact on abiotic stress tolerance. GS is also the target enzyme of the broad spectrum herbicide Glufosinate (active ingredient: phosphinothricin). In this study, we investigated the effect of concurrent overexpression of the rice cytosolic GS1 (*OsGS1;1*) and chloroplastic GS2 (*OsGS2*) genes in transgenic rice on its tolerance to abiotic stresses and the herbicide Glufosinate. Our results demonstrate that the co-overexpression of *OsGS1;1* and *OsGS2* isoforms in transgenic rice plants enhanced its tolerance to osmotic and salinity stress at the seedling stage. The transgenic lines maintained significantly higher fresh weight, chlorophyll content, and relative water content than wild type (*wt*) and null segregant (*ns*) controls, under both osmotic and salinity stress. The *OsGS1;1/OsGS2* co-overexpressing transgenic plants accumulated higher levels of proline but showed lower electrolyte leakage and had lower malondialdehyde (MDA) content under the stress treatments. The transgenic lines showed considerably enhanced photosynthetic and agronomic performance under drought and salinity stress imposed during the reproductive stage, as compared to *wt* and *ns* control plants. The grain filling rates of the transgenic rice plants under reproductive stage drought stress (64.6 ± 4.7%) and salinity stress (58.2 ± 4.5%) were significantly higher than control plants, thereby leading to higher yields under these abiotic stress conditions. Preliminary analysis also revealed that the transgenic lines had improved tolerance to methyl viologen induced photo-oxidative stress. Taken together, our results demonstrate that the concurrent overexpression of *OsGS1;1* and *OsGS2* isoforms in rice enhanced physiological tolerance and agronomic performance under adverse abiotic stress conditions, apparently acting through multiple mechanistic routes. The transgenic rice plants also showed limited tolerance to the herbicide Glufosinate. The advantages and limitations of glutamine synthetase overexpression in crop plants, along with future strategies to overcome these limitations for utilization in crop improvement have also been discussed briefly.

## Introduction

Glutamine synthetase (GS; L-glutamate-ammonia ligase; EC 6.3.1.2) is a key enzyme involved in nitrogen (N) metabolism of plants, as it catalyzes the critical incorporation of inorganic ammonium into glutamine in an ATP-dependent manner (Miflin and Habash, 2002; Bernard and Habash, 2009). GS assimilates ammonia (NH${}_{4}^{+}$), a cytotoxic and reactive metabolite, produced from the fixation of atmospheric N, and or during direct nitrate or ammonia uptake from soil (Hirel and Lea, 2001). GS is also responsible for the re-assimilation of NH${}_{4}^{+}$, produced during various cellular metabolic processes including, photorespiration and protein degradation, which are further enhanced particularly during stress or senescence (Bernard and Habash, 2009). Along with glutamate synthase (GOGAT; EC 1.4.7.1 and EC 1.4.1.14), GS takes part in the GS/GOGAT cycle which is the focal point of N metabolism in higher plants. The amino acids glutamine and glutamate thus produced, are used to synthesize all other organo-nitrogen compounds including nucleotides, chlorophyll, and also other amino acids like proline etc. (Forde and Lea, 2007; Bernard and Habash, 2009). The efficient functioning of GS is crucial, as the buildup of NH${}_{4}^{+}$ can cause cell death and severe damage to plant tissues (Wild and Manderscheid, 1984; Tachibana et al., 1986). Two isoforms of GS, the cytosolic GS1, and the chloroplastic GS2 are generally present in higher plants. The smaller cytosolic isoform GS1 is responsible for the primary assimilation of inorganic N availed from the soil in the form of nitrate or ammonia, and the re-assimilation of NH${}_{4}^{+}$ released by protein degradation in senescing leaves (Bernard and Habash, 2009). Whereas, the larger chloroplast localized isoform, GS2, is responsible for re-assimilation of NH${}_{4}^{+}$ released during photorespiration and nitrate (NO${}_{3}^{-}$) reduction in plastids (Wallsgrove et al., 1987; Leegood et al., 1995; Lam et al., 1996). In most plants, a multigene family encodes the cytosolic GS1, while only a single gene encodes the chloroplastic GS2. In rice, one gene encodes the plastidic GS2 (*OsGS2*) and three encode cytosolic GS1 (*OsGS1;1, OsGS1;2*, and *OsGS1;3*). *OsGS1;1* is ubiquitous but expressed more in the shoot and stem, *OsGS1;2* is expressed mostly in the root, *OsGS1;3* is limited to the spikelets, whereas the *OsGS2* isoform is abundant in the leaf (Tabuchi et al., 2005). Due to its central role in N metabolism, GS is considered a prime target for transgenic approaches to increase nitrogen use efficiency (NUE) and yield, which is paramount in achieving sustainability in agriculture and ensuring food security for our burgeoning population (Brauer and Shelp, 2010; Swarbreck et al., 2011). Hence most studies have largely focused attention on the impact of transgenic overexpression of GS on the parameters such as yield and NUE in various plants such as tobacco (Migge et al., 2000; Fuentes et al., 2001; Oliveira et al., 2002; Wang et al., 2013), Arabidopsis (Zhu et al., 2014, 2015), maize (Martin et al., 2006); poplar (Gallardo et al., 1999; Fu et al., 2003; Jing et al., 2004; Man et al., 2005), sorghum (Urriola and Rathore, 2015); wheat (Habash et al., 2001), and rice (Cai et al., 2009; Brauer et al., 2011; Bao et al., 2014).

However, genes involved in N metabolism, including GS, are also known to be significantly modulated during various stress responses in different plants (Wang et al., 2012; Goel and Singh, 2015). The expression and activity of GS isoforms have been reported to be modulated in various plants in response to abiotic stresses like drought (Bauer et al., 1997; Nagy et al., 2013; Singh and Ghosh, 2013; Yousfi et al., 2015; Cheng et al., 2016), cold (Lu et al., 2005; Kwon et al., 2007), salinity (Silveira et al., 2001, 2003; Yan et al., 2005; Ouyang et al., 2007; Teixeira and Pereira, 2007; Wang et al., 2007, 2012), and metal toxicity (Chaffei et al., 2004; Rana et al., 2008). Several studies on the GS enzyme have postulated its role in improving tolerance to various abiotic stresses. For example, a comparative study on the expression and activity of various GS isoforms in rice under drought stress inferred that a relatively maintained *OsGS2* level and the over-expression of *OsGS1;1* might contribute to the enhanced drought tolerance characteristics of the drought tolerant rice cultivar Khitish (Singh and Ghosh, 2013). In addition, gene expression analysis under salinity and drought stress between contrasting durum wheat genotypes showed that the most tolerant genotype exhibited the highest GS activity and had enhanced expression of both GS1 and GS2 isoforms under stress conditions as compared to the control plants (Yousfi et al., 2015). Nagy et al. (2013) observed that drought tolerant wheat genotypes maintained higher GS activity in the flag leaf under drought stress than sensitive cultivars. Furthermore, a comprehensive QTL analysis in potato revealed that the cytosolic GS is essential for improving photosynthetic efficiency and water use efficiency (WUE). It was observed that the GS activity was more enhanced in the high WUE bulk population than in the low WUE bulk population (Kaminski et al., 2015). On similar lines, a recent proteomic study of wheat cultivars under drought stress found that the chloroplastic GS2 was significantly up-regulated in a drought tolerant cultivar as compared to the sensitive cultivar (Cheng et al., 2016).

Hence, several lines of evidence have propounded the ability of GS to confer enhanced tolerance and higher yields of crops under various abiotic stresses. However, only a few transgenic overexpression studies of GS isoforms have studied its impact on tolerance to abiotic stresses. For instance, overexpression of a cytoplasmic GS1 gene from conifer in transgenic poplar trees was observed to confer enhanced tolerance to drought stress (El-Khatib et al., 2004), while transgenic rice lines overexpressing the chloroplastic *OsGS2* gene displayed enhanced salinity tolerance (Hoshida et al., 2000). In addition, the constitutive overexpression of the cytosolic *OsGS1;1* gene in rice improved tolerance to oxidative stress induced by the heavy metal cadmium (Lee et al., 2013).

GS is also the target enzyme of the widely used post emergent broad spectrum herbicide Glufosinate (active ingredient: L-Phosphinothricin/PPT; common trade name: Basta™). Phosphinothricin (PPT) is a structural analog of the substrate of GS *viz*. glutamate, and occupies the substrate pocket of the enzyme and blocks glutamate binding to GS (Gill and Eisenberg, 2001). Inhibition of the GS enzyme by PPT causes the buildup of ammonia in plant cells, inhibition of amino acid synthesis and inhibition of photosynthesis, which ultimately leads to the death of the plants (Donn and Köcher, 2002). Several studies have observed that transgenic overexpression of GS confers tolerance to PPT in plants like poplar (Pascual et al., 2008) and rice (Cai et al., 2009). Also, the co-overexpression of a cytosolic GS1 and chloroplastic GS2 from pea was reported to confer tolerance to PPT in wheat and rice transgenics (Huang et al., 2005; Sun et al., 2005).

Since GS is a potential target in multiple avenues of transgenic crop improvement including yield, NUE, abiotic stress tolerance and herbicide resistance, the co-overexpression of multiple GS isoforms through gene stacking was considered expedient to study its effects on these multiple areas. The effect of *OsGS1;1/OsGS2* co-overexpression on yield and NUE of rice under varying N regimens is currently being investigated, and will be reported separately. In this study, through an *in vitro* gene pyramiding approach utilizing a Multi-Round Gateway cloning technology, we were able to concurrently overexpress the cytosolic GSl;1 (*OsGS1;1*) and chloroplastic GS2 (*OsGS2*) in transgenic rice. Its impact on abiotic stresses tolerance and resistance to the herbicide Glufosinate (phosphinothricin) is discussed here within.

## Materials and methods

### *In vitro* gene pyramiding of rice GS isoforms into plant transformation vector and generation of transgenic rice

**Figure 1:**
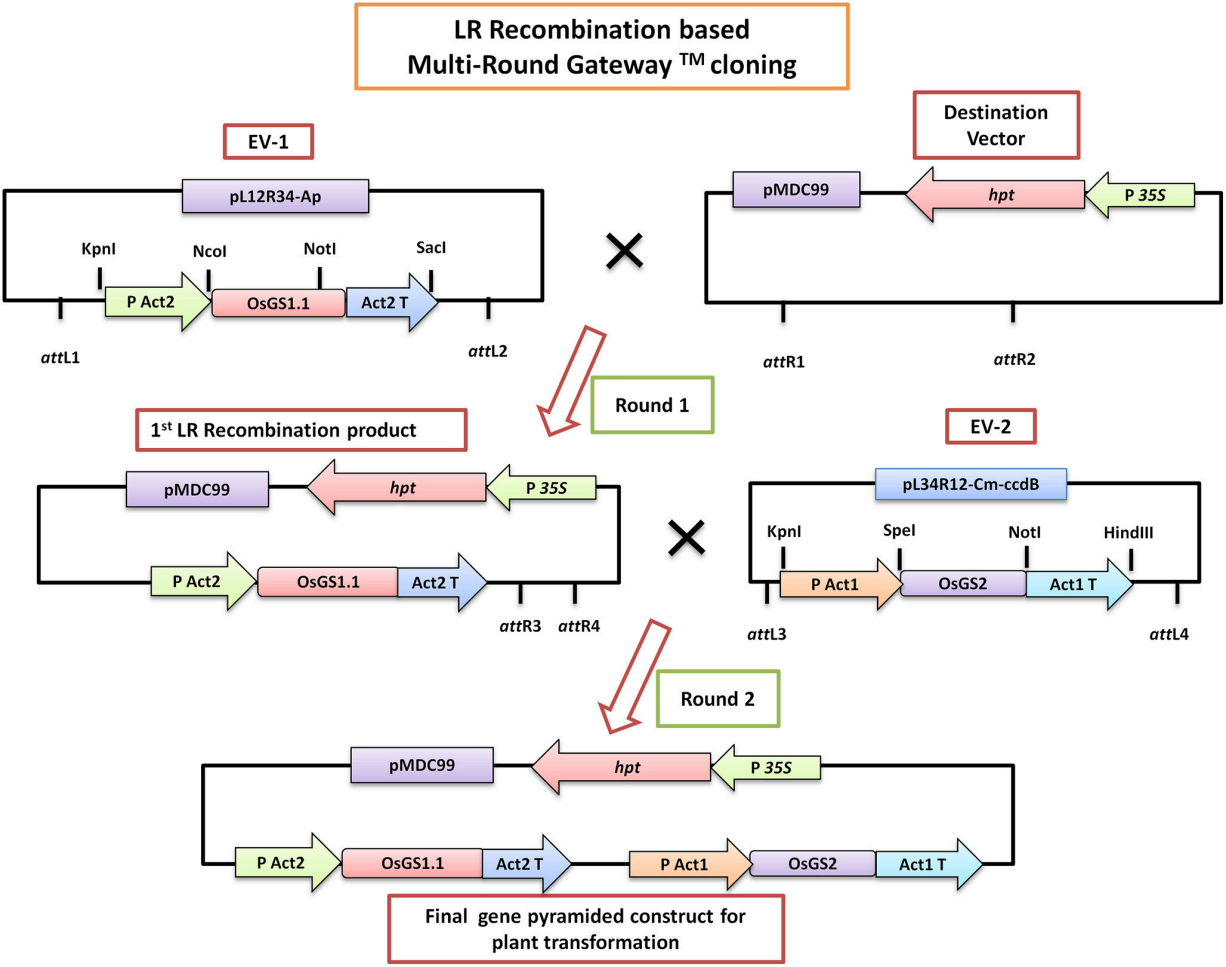
A simplified schematic diagram of the LR-Recombination based Multi-Round Gateway™ technology used for *in vitro* pyramiding of the *OsGS1;1* and *OsGS2* genes. The *OsGS1;1* gene was cloned into the Entry vector 1 (EV-1) by restriction enzyme based cloning under the rice Actin 2 promoter (PAct2) and Act2 terminator (Act2T) whereas the *OsGS2* gene was cloned into the entry vector 2 (EV-2) under the rice Actin 1 promoter (PAct1) and Act1 terminator (Act1T). In the first round of LR cloning, EV-1 was recombined with the destination vector (pMDC99) to obtain the first LR recombined product. Subsequently, EV-2 was recombined with the first LR product in a second round of LR cloning to obtain the final gene pyramided pMDC99 construct containing both *OsGS1;1* and *OsGS2* genes to be used for plant transformation (For a detailed scheme see Chen et al., 2006).

Gene specific primers were designed based on the sequence of rice cytosolic GS1;1 (*OsGS1;1*; LOC_Os02g50240) and rice chloroplastic GS2 (*OsGS2*; LOC_Os04g56400) available in the Rice Genome Annotation database (Supplementary Table1). The full-length coding sequences of *OsGS1;1* (1113 bp) and *OsGS2* (1287 bp) were amplified by PCR from rice (*Oryza sativa* L. ssp japonica cv Nipponbare) cDNA using a high fidelity DNA polymerase (KOD plus, Toyobo, Japan) and cloned into pCR-4-TOPO vector (Invitrogen, USA). Following verification by sequencing, the *OsGS1;1* gene was sub-cloned into a Gateway compatible entry vector EV-1 (pL12R34-Ap) in between the rice Actin 2 (*OsAct2*) promoter and rice Actin 2 (*OsAct2*) terminator, whereas the *OsGS2* gene was cloned into the Gateway compatible EV-2 (pL34R12-Cm-ccdB) vector under the rice Actin 1 (*OsAct1*) promoter and rice Actin 1 (*OsAct1*) terminator. The plant expression cassettes of rice cytosolic GS (*OsAct2* promoter: *OsGS1;1*: *OsAct2* terminator) and plastidic GS (*OsAct1* promoter: *OsGS2*: *OsAct1* terminator) from the entry vectors were sequentially cloned into a Gateway compatible destination vector (pMDC99) for plant transformation using a Multi-Round LR recombinase mediated Gateway™ (Invitrogen, USA) cloning process as described previously (Chen et al., 2006) (Figure 1). The pMDC99 vector contains the hygromycin resistance gene hygromycin phosphotransferase (*hpt*) as plant selection marker (Curtis and Grossniklaus, 2003). The *in vitro* gene pyramided construct containing both *OsGS1;1* and *OsGS2* under constitutive rice actin promoters (pMDC99- *OsAct2* P: *OsGS1*;*1*: *OsAct2* T:: *OsAct1* P: *OsGS2*: *OsAct1* T) was transformed into rice (*O*.*sativa* ssp Japonica) cultivar Nipponbare through *Agrobacterium* mediated transformation using a protocol described earlier (Ravikumar et al., 2014). Briefly, twenty-one-day-old scutellum-derived embryogenic calli were infected with *Agrobacterium* EHA105 strain harboring the above construct and co-cultivated for 2 days on Chu-N6 medium (Duchefa, Germany) supplemented with acetosyringone (200 μM). The transformed calli were selected on Chu-N6 medium supplemented with 50 mg/L Hygromycin (Invitrogen, USA) and 1 mg/L PPT (Duchefa, Germany). Secondary calli that developed after four rounds of selection were transferred to regeneration media for the development of shoots. The regenerated shoots were transferred to rooting media. The transgenic *OsGS1;1/OsGS2* co-overexpressing primary transformants (T_0_) were transferred to soilrite for hardening and then transferred to soil pots in a greenhouse maintained at 28 ± 2°C temperature and relative humidity of 70 ± 5%.

### Molecular confirmation of transgene insertion

Putative T_0_ transgenics seeds were screened by germinating them on $\frac{1}{2}$ Murashige and Skoog media containing 50 mg/L hygromycin, followed by PCR confirmation using *hpt, OsGs1;1* and *OsGS2* screening primers (Supplementary Table 1). Transgenic plants were progressed to the T_2_ generation in the greenhouse following standard agronomic practices. T_2_ generation plants were again verified by PCR using *hpt, OsGS1;1*: *OsAct2T* and *OsGS2*: *OsAct1T* screening primers (Supplementary Table 1). Null segregants (*ns*) (azygous lines from the T_1_ generation of transgenics) identified using PCR were used as controls along with wild type (*wt*) rice plants to account for probable *in vitro* regeneration and transformation effects. Transgene insertion and copy number assessment was done by Southern blot analysis in T_2_ generation plants using DIG non-radioactive nucleic acid labeling and detection system (Roche, QC, Canada) as per the protocol followed by Manna et al. (2016). Briefly, 20 μg of genomic DNA of control and transgenic rice lines was digested with *HindIII* and genomic DNA fragments were separated on a 0.8% agarose gel, blotted onto Hybond™ N^+^ nylon membrane (GE Healthcare Limited, UK), and subsequently hybridized with a 900 bp *hpt* gene specific probe. The probe was synthesized through PCR using *hpt* gene specific primers (Supplementary Table 1) labeled with DIG (PCR DIG Probe Synthesis Kit, Roche, Germany). The blot was washed and detected according to the manufacturer's instructions (DIG High Prime DNA Labeling and Detection Starter Kit II, Roche, Germany).

Gene expression analysis by semi-quantitative RT-PCR was carried out using *OsSG1;1* and *OsGS2* RT-PCR specific primers (Supplementary Table 1) using standard protocols. The eEF-1α gene from rice was used as endogenous reference gene for normalizing the relative expression (Jain et al., 2006). The RT-PCR amplified products were resolved on a 1.2% agarose gel. Densitometric analysis of the bands was performed using the Image J software, to quantify relative transcript expression levels (Schneider et al., 2012).

### Total GS activity assay

For total GS enzyme activity assay, 2-week-old seedlings of T_2_ transgenic plants and wild type Nipponbare (*wt*) controls were homogenized in a GS extraction buffer (15 mL/g fresh weight) containing 50 mM Tris-HCl (pH 8), 5 mM MgC1_2_, 5% (w/v) insoluble PVP, and 15% glycerol. The crude extract was filtered through five layers of gauze and the filtrate was centrifuged at 12,000 rpm for 30 min at 4°C (Singh and Ghosh, 2013). The total protein in the crude supernatant was quantified by Bradford assay, and used for both assay of GS activity and immunoblotting analyses of GS1 and GS2.

Total GS activity was measured using the semi-biosynthetic assay protocol modified from Singh and Ghosh (2013) by quantifying the formation of γ-glutamylhydroxamate. Briefly, the reaction mixture of total 1 mL volume consisted of 50 mM Tris–HCl (pH 7.5), 250 mM glutamate, 20 mM ATP, 10 mM hydroxylamine hydrochloride, 20 mM MgCl_2_, and 200 μL of crude extract. The reaction mixture was kept at 37°C for 20 min and terminated by adding 2 mL of FeCl_3_ reagent (0.67 M FeCl_3_, 0.37 M HCl and 20% (w/v) tri-chloroacetic acid). The reaction mixture was incubated at room temp for 10 min for color development. The reaction mixture was then centrifuged at 4,000 g at room temperature for 10 min, and 1 mL of the supernatant was transferred into a quartz cuvette and the absorbance measured via a spectrophotometer (Ultrospec 2100 pro, Amersham Biosciences, UK) at 540 nm. Values of GS activity were extrapolated from a standard calibration curve made from different known concentrations of γ-glutamylhydroxamate (Sigma-Aldrich, USA). One unit of GS activity represents 1 μmol of γ-glutamylhydroxamate produced in 20 min.

Immunoblotting was done using a protocol modified from Kamachi et al. (1992) using recombinant GS specific antibodies that detect both GS1 and GS2 isoforms (Ishiyama et al., 2004). Briefly, 5 μg total protein from *wt* and transgenic plants extracted as described above, were separated on 12.5% (w/v) SDS PAGE and blotted onto a PVDF membrane (Millipore, USA) using as Mini transblot electrophoretic cell (Biorad, USA). The blot was incubated in blocking solution (5% non-fat dry milk in PBS) for 1 h. The blot was then washed with PBS containing 0.1% Tween-20 (PBST) for three times at 10 min intervals. Following incubation with recombinant antibody at 1:1,000 dilution in blocking solution, the blot was washed with PBST three times at 10 min intervals. Subsequently, the blot was incubated with alkaline phosphatase conjugated anti-rabbit IgG secondary antibody (Sigma, USA) (1:5,000 dilution in PBS) for 1 h, and then was washed three times with PBS containing 0.3% Tween-20 and again three times with PBST at 10 min intervals. Bands were detected using a ready to use BCIP/NBT solution (Sigma, USA) in a dark room until color development and thereafter the reaction was stopped by washing in PBS. The bands were relatively quantified using densitometric analysis of their intensities using the Image J software (Schneider et al., 2012).

### Plant growth conditions and stress treatments

The surface sterilized *OsGS1;1/OsGS2* co-overexpressing transgenic rice seeds along with seeds of wild type (*wt*) and null segregant (*ns*) plants (both hereafter referred to as “control plants”) were sown on $\frac{1}{2}$ Murashige and Skoog medium or sterile germination paper rolls, and then transferred to a growth chamber at 28°C under a 16-h-light/8-h dark photoperiod and 70% relative humidity. For various analyses, both *OsGS1;1/OsGS2*-overexpressing transgenic and control rice seeds were germinated on moist paper rolls and then transferred to YS hydroponic culture solution (Yoshida nutrient solution; Yoshida et al., 1976) at the two-leaf stage. The culture solution was replaced once every 2 days and pH set to 5.5. For stress treatments, 2-week-old seedlings were transferred to YS supplemented with 150 mM NaCl (EC ~ 14 dS/m) for imposing salinity stress and 15% PEG (MW 6000) for inducing osmotic or physiological drought stress. The plants were grown for 7 days to assess phenotypic variation in physiological tolerance. In a separate experiment, 2-week-old seedlings of transgenic and control plants were grown in YS supplemented with 20% PEG (MW 6000) or 200 mM NaCl (EC ~ 19 dS/m) for 4 days followed by recovery in normal YS for 4 days to determine tolerance to high drought and salinity stresses. Various biochemical and physiological parameters were assessed after 2 days of stress treatments. The electrical conductivity (EC) of the hydroponic solutions was measured using an electrical conductivity meter (WTW Cond 315i, Germany) according to manufacturer's instructions.

For assessing tolerance to drought at reproductive stage, transgenic plants along with control plants were grown in 500 mL pots filled with a 1:1 mix of topsoil and manure compost (quartered and mixed to homogeneity), and placed in water-filled trays to simulate paddy conditions. The rice plants were grown to panicle initiation stage under optimum agronomic conditions in a green house at (16-h-light/8-h-dark cycles) at 28 to 30°C. Drought stress was imposed by withdrawing water using the gravimetric method for a period of 12 days post panicle initiation, followed by recovery by regular irrigation until maturity to assess yield parameters (protocol modified from Ambavaram et al., 2014). For assessing the impact of salinity stress on growth and yield parameters, ~2-month-old *OsGS1;1/OsGS2* co-overexpressing transgenic rice plants along with controls were subjected to moderate salinity stress by irrigating the pots every fortnight with deionized water supplemented with 50 mM NaCl (EC ~ 6 dS/m) (protocol modified from Tripathi et al., 2016). The treatment was continued till initiation of the booting stage after which the plants were recovered and grown until maturity, and thereafter their agronomic performance assessed. Untreated control pots were irrigated with normal deionized water.

For oxidative stress treatment assays, leaf strips of 2 cm length and uniform width from flag leaf of mature T_2_ generation transgenic lines along with the control plants were incubated in 10 μM methyl viologen (MV; paraquat) for 6 h under dark at 28°C to allow diffusion of MV into the tissue and then exposed to 4 h of sunlight (Mahanty et al., 2012). The total chlorophyll content after MV treatment was determined spectrophotometrically following the protocol of Hiscox and Israelstam (1979). Oxidative stress induced generation of H_2_O_2_ after treatment with MV was detected by incubating the leaf strips with 3,3-diaminobenzidine (DAB) (pH 3.8), until a reddish-brown color developed (Thordal-Christensen et al., 1997). *In-vivo* generation of O${}_{2}^{-}$ in leaves after MV treatment was detected by staining with 1% nitro blue tetrazolium (NBT) in 10 mM sodium phosphate buffer, until a purple-blue color was observed (Lin et al., 2009). After NBT and DAB staining, the chlorophyll of the treated samples was removed by boiling in 95% ethanol, and the images of leaf strips were photographed.

To assess tolerance to PPT, mature leaves of both *wt* and T_2_ transgenic rice plants were painted with solutions of 0.5, 1, and 2% Basta (v/v) (Bayer, 13.5% ai) supplemented with 0.01% Tween-20. The leaves were scored for tolerance after 5 days of treatment based on the degree of leaf burning, bleaching and necrosis (Tsai et al., 2006). In addition, T_2_ transgenic and *wt* plants at four-six leaf stage were sprayed with a 0.5% (v/v) solution of Basta. After 1 week, the growth of the plants was assessed and survival rates were calculated.

### Assessment of various biochemical and physiological parameters

Total chlorophyll contents of untreated and stressed seedlings were measured spectrophotometrically following the protocol of Hiscox and Israelstam (1979). Relative chlorophyll content of plants under reproductive stage stress experiments was measured on the third fully opened leaf from the top using a SPAD 502 portable chlorophyll meter (Minolta, Japan). RWC (Relative Water Content) was measured according to the protocol of Barrs and Weatherley (1962). Electrolyte leakage measurements after stress treatments were performed as previously described by Sairam et al. (2005). Measurement of proline was performed according to the protocol of Bates et al. (1973). Estimation of lipid peroxidation was done by determining the amount of MDA (malondialdehyde) using the TBARS (thiobarbituric acid-reactive-substances) assay following the protocol of Hodges et al. (1999). Mean NH${}_{4}^{+}$ liberation with and without PPT application was determined by a modified Berthelot reaction assay given by (Rasco-Gaunt et al., 1999). The plant traits and yield components of rice namely plant height (cm), number of panicles, grain filling rate (%), and grain yield per plant (g), were measured as described by Yoshida et al. (1976).

### Measurement of photosynthetic parameters

Photosynthetic gas exchange parameters were measured in the morning (9:00–11:00 a.m.), on flag leaves, using an Infrared Gas Analyzer (IRGA; LI-6400XT portable photosynthesis system equipped with a LI-6400-40 Leaf Chamber Fluorometer, LICOR, Lincoln NB). The measurements were made at a CO_2_ concentration of 400 μmol mol^−1^, PPFD of 1200 μmol m^−2^ s^−1^ and a chamber temperature of 30°C. The chlorophyll fluorescence (F_v_/F_m_) during various stress conditions were measured after dark adaptation for 30 min following the protocol outlined by Murchie and Lawson (2013). The measurements of photosynthetic parameters for drought stress treated plants were made after 4 days of recovery following 12 days of drought stress treatment. Measurements for salinity stress treatments were taken after 7 days of recovery following final salinity stress treatment. All the measurements were repeated five times on fully expanded flag leaves of control and transgenic plants and the means ± SD are represented.

### Statistical analyses

All data represented are means ± SD (*n* = 3). Data were analyzed using ANOVA and *post-hoc* Tukey-Kramer multiple comparisons tests to assess significance among means using the statistical tools available in GraphPad Prism 6 ^TM^ software.

## Results

### Generation of *in vitro* pyramided construct, generation, and confirmation of rice transgenics

**Figure 2:**
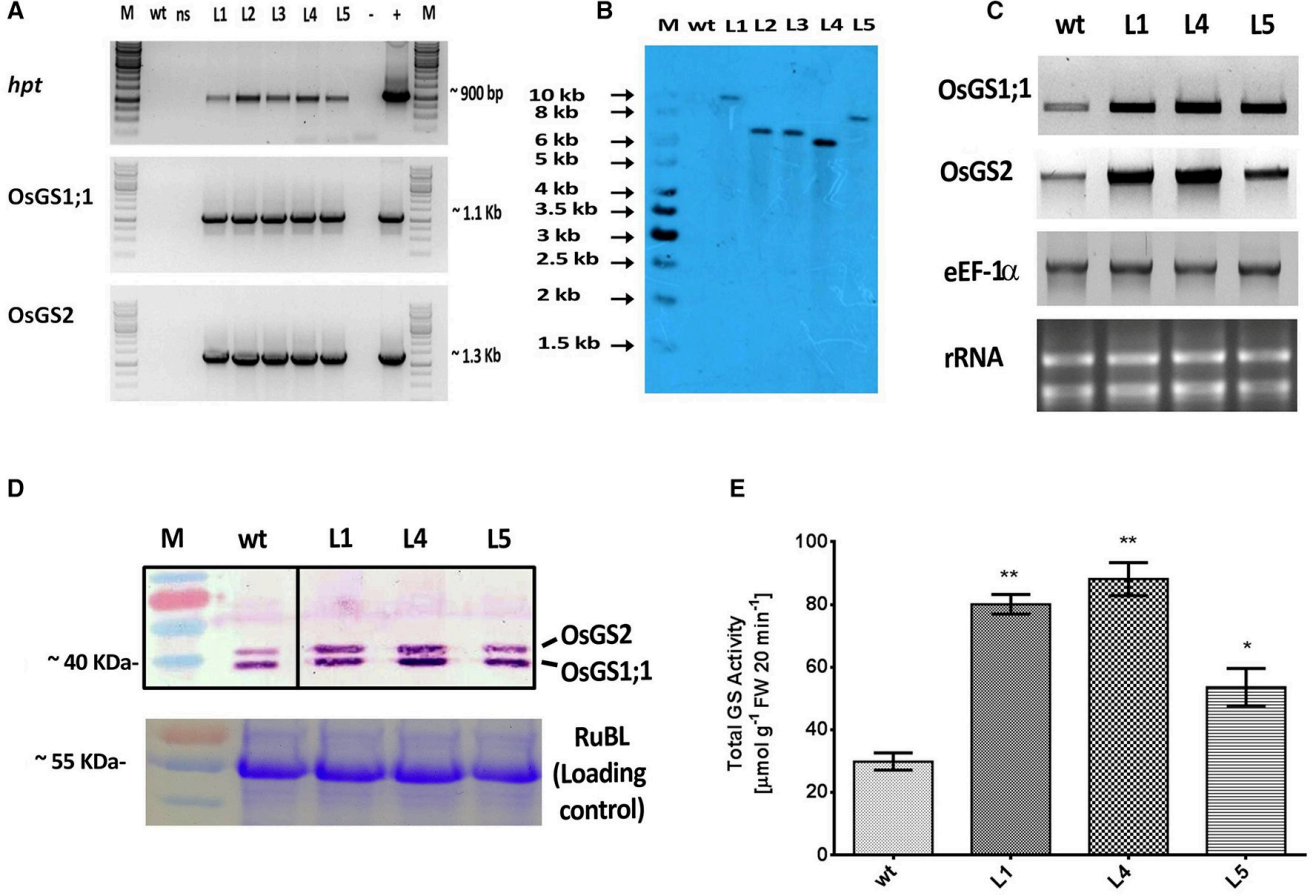
Molecular and biochemical analysis of transgenic rice lines co-overexpressing *OsGS1;1* and *OsGS2*.**(A)** PCR amplification of hygromycin phosphotransferase (*hpt*), *OsGS1;1* and *OSGS2* genes using specific primers in wild type (*wt*), null segregant (*ns*), and five positive T_2_ transgenic lines (L1-L5). M: 1Kb DNA ladder (+): positive PCR control (pMDC99) and (–) water blank. **(B)** Southern blot analysis of *wt* and five T_2_ transgenic lines (L1, L2, L3, L4, and L5), probed with *hpt* gene probe showing single copy insertion. **(C)** Semi quantitative RT-PCR showing overexpression of *OsGS1;1* and *OsGS2* in transgenic lines (L1, L4, and L5) as compared to *wt*. The rice eEF1α gene was used as a reference gene and rRNA was used as loading control. **(D**; top panel) Immunoblot analysis of three transgenic rice lines (L1, L4, and L5) and *wt* using a recombinant antibody which detects both OsGS1;1 and OsGS2 isoforms (black lines separate spliced regions from same blot) (**D**; bottom panel) Coomassie blue stained Rubisco large subunit (RubL) was used as loading control. **(E)** Total GS activity of three transgenic rice lines (L1, L4, and L5) in comparison to *wt* as assayed by a modified semi-biosynthetic assay (Singh and Ghosh, 2013). One unit of GS activity represents 1.0 μmol of γ-glutamylhydroxamate produced in 20 min. Asterisks above bars indicate significant differences from *wt* (* at *p* ≤ 0.05 and ** at *p* ≤ 0.01).

The rice GS isoforms *OsGS1;1* and *OsGS2* were successfully PCR amplified from rice cDNA. The rice GS1;1 encoding sequence was successfully cloned between the *OsAct2* promoter and *OsAct2* terminator as a plant expression cassette in EV-1 while the rice GS2 encoding sequence was cloned between the rice *OsAct1* promoter and *OsAct1* terminator in EV-2 to constitutively overexpress the rice cytosolic and plastidic GS isoforms. Both the expression cassettes were *in vitro* pyramided on to a single plant transformation vector (pMDC99) using the Multi-Round Gateway cloning technology (Figure 1). The recombinant construct was then transformed into EHA 105 strain of *Agrobacterium* by electroporation and used for rice transformation. A total of around 150 putative T_0_ transgenic rice lines were generated. Screening of the putative transgenics to check the integration of *hpt, OsGS1;1* and *OsGS2* genes into rice genome showed that more than 80% of the putative transformants were PCR positive and showed integration of all the transgenes. The PCR analysis of five selected positive T_2_ transgenics (L1, L2, L3, L4, and L5) showed the expected ~1 Kb DNA fragment with *hpt* primer set, ~1.1 kb DNA fragment with *OsGS1;1*: *OsAct2* terminator primer set, and ~1.3 kb fragment with *OsGS2*: *OsAct1* terminator primer set respectively from the transgenic rice genomic DNA, whereas no such DNA fragments were amplified from untransformed wild type (*wt*) rice genomic DNA (Figure 2A). Southern analysis of selected T_2_ transgenic lines probed with *hpt* gene sequence showed a single hybridization band with a distinct pattern for each transgenic line suggesting independent single copy integration of the transgene in each transgenic event (Figure 2B). The untransformed *wt* plants did not show any hybridization signal. Three independent T_2_ transgenic lines (L1, L4, and L5), which showed PCR positive amplification for *hpt, OsGS1;1* and *OsGS2* transgene cassettes and single copy positive southern hybridization were selected for semi quantitative RT-PCR analysis. All the selected transgenic lines (L1, L4, and L5) showed higher expression of *OsGS1;1* and *OsGS2* transcripts compared to *wt* controls as quantified by densitometric analysis of semi-quantitative RT-PCR bands (Figure 2C). Transgenic line L1 had 2.5-fold increased *OsGS1;1* and 2.7-fold increased *OsGS2* expression levels while L4 had 3.1-fold increased *OsGS1;1* expression and 2.9-fold increased *OsGS2* expression. Whereas, transgenic line L5 showed only 2-fold increase in expression of *OsGS1;1* and *OsGS2* compared to *wt* controls (Supplementary Figure S1). The immunoblotting analysis also showed the increased accumulation of OsGS1;1 and OsGS2 protein in all the selected transgenic lines (Figure 2D). An immunopositive 39 kDa polypeptide signal which corresponded to the OsGS1;1 isoform and a 42 kDa polypeptide signal which corresponded to the chloroplastic OsGS2 isoform were observed (Figure 2D). Densitometric analysis of the immunopositive signals using the Image J software showed that transgenic lines L1 and L4 had around 3-fold higher OsGS2 protein content and 2-fold increase in OsGS1;1 protein content in total soluble protein extracts from the transgenic rice seedlings (Supplementary Figure S2). Total GS enzyme activity varied between the selected transgenic lines with L1 and L4 having around 4-folds and L5 having close to 2-fold higher activities as compared to *wt* controls (Figure 2E). Our results demonstrate that the increase in expression of the GS isoforms in the *OsGS1;1/OsGS2* co-overexpressing transgenic Nipponbare rice, led to a corresponding increase in GS protein as well as total GS enzymatic activity (Figures 2C–E). However, it has been reported that higher content of GS protein did not always correspond to higher enzymatic activities of GS in indica rice varieties, and this was attributed to post translational regulation (Obara et al., 2000).

### Concurrent *OsGS1;1* and *OsGS2* overexpression in rice confers tolerance to osmotic and salinity stress at seedling stage

**Figure 3:**
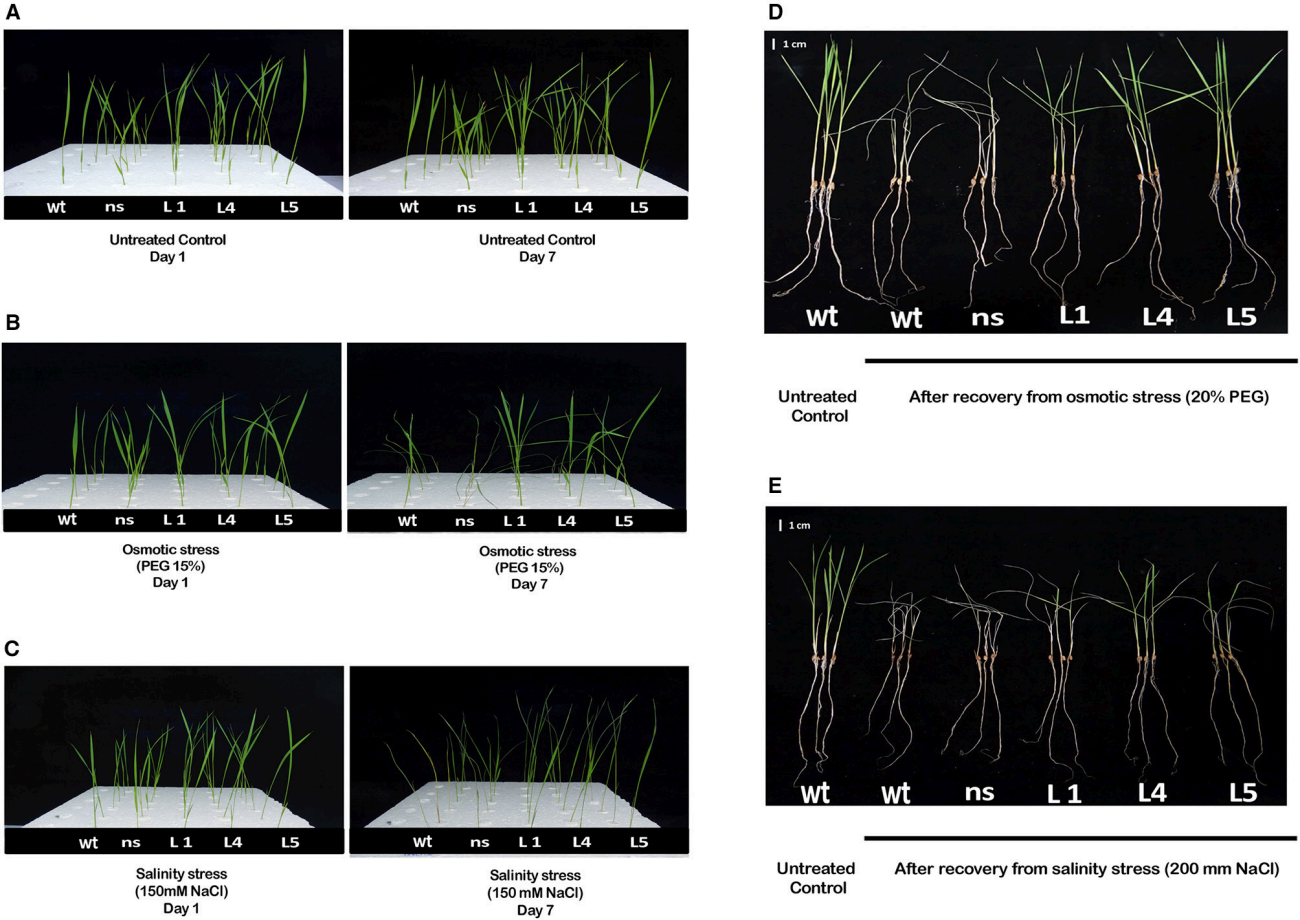
Phenotype of *OsGS1;1/OsGS2* co-overexpressing transgenic rice under moderate osmotic and salinity stress at seedling stage. Phenotype of 2-week-old seedlings of *wt, ns* and three transgenic rice lines (L1, L4, and L5), grown hydroponically in **(A)** normal Yoshida solution (untreated control) or **(B)** Yoshida solution supplemented with 15% PEG (osmotic stress) or **(C)** 150 mM NaCl (EC ~ 12 dS/m) (salinity stress) before and after 7 days of treatment. Visual phenotypic variation amongst seedlings of *wt, ns* and three transgenic rice lines (L1, L4, and L5), following 4 days of recovery after being grown hydroponically for 4 days in **(D)** Yoshida solution supplemented with 20% PEG (osmotic stress) or **(E)** 200 mM NaCl (EC ~ 19 dS/m) (salt stress). Scale bar = 1 cm.

Two-week-old transgenic T_2_ lines, *wt* and *ns* seedlings were grown hydroponically either in untreated YS solution (untreated control) or grown in YS supplemented with 150 mM NaCl (EC~12 dS/m) (salinity stress) or in YS supplemented with 15% PEG (osmotic/physiological drought stress) to study the physiological tolerance of *OsGS1;1/OsGS2* overexpressing transgenic rice lines to abiotic stresses. The transgenic, *wt* and *ns* seedlings grew well and produced new leaves in the untreated control set (Figure 3A). However, when grown in YS supplemented with either 15% PEG or 150 mM NaCl, there were apparent differences in visible symptoms between *OsGS1;1/OsGS2* co-overexpressing transgenic rice seedlings and control seedlings after 7 days of treatments (Figures 3B,C). In the presence of 15% PEG, the *wt* and *ns* control plants showed wilting and leaf rolling within 7 days, whereas the transgenic seedlings were able to maintain turgidity and had no symptoms of leaf rolling or wilting (Figure 3B). The *OsGS1;1/OsGS2* co-overexpressing transgenic plants were able to survive 15% PEG induced osmotic stress up to 12 days while the control plants underwent severe wilting or died (Supplementary Figure S3). Under salinity stress (150 mM NaCl), both transgenic and control plants showed retarded seedling growth and reduced leaf size compared to the corresponding seedlings grown in untreated YS. However, visible symptoms like leaf tip burning, necrosis, yellowing, wilting and leaf rolling were observed in *wt* and *ns* seedlings, whereas such symptoms were not very significant in the transgenic seedlings (Figure 3C). Overall, the *OsGS1;1/OsGS2* co-overexpressing transgenic seedlings displayed better growth and increased tolerance to salinity stress in comparison to both *wt* and *ns* control plants.

**Figure 4:**
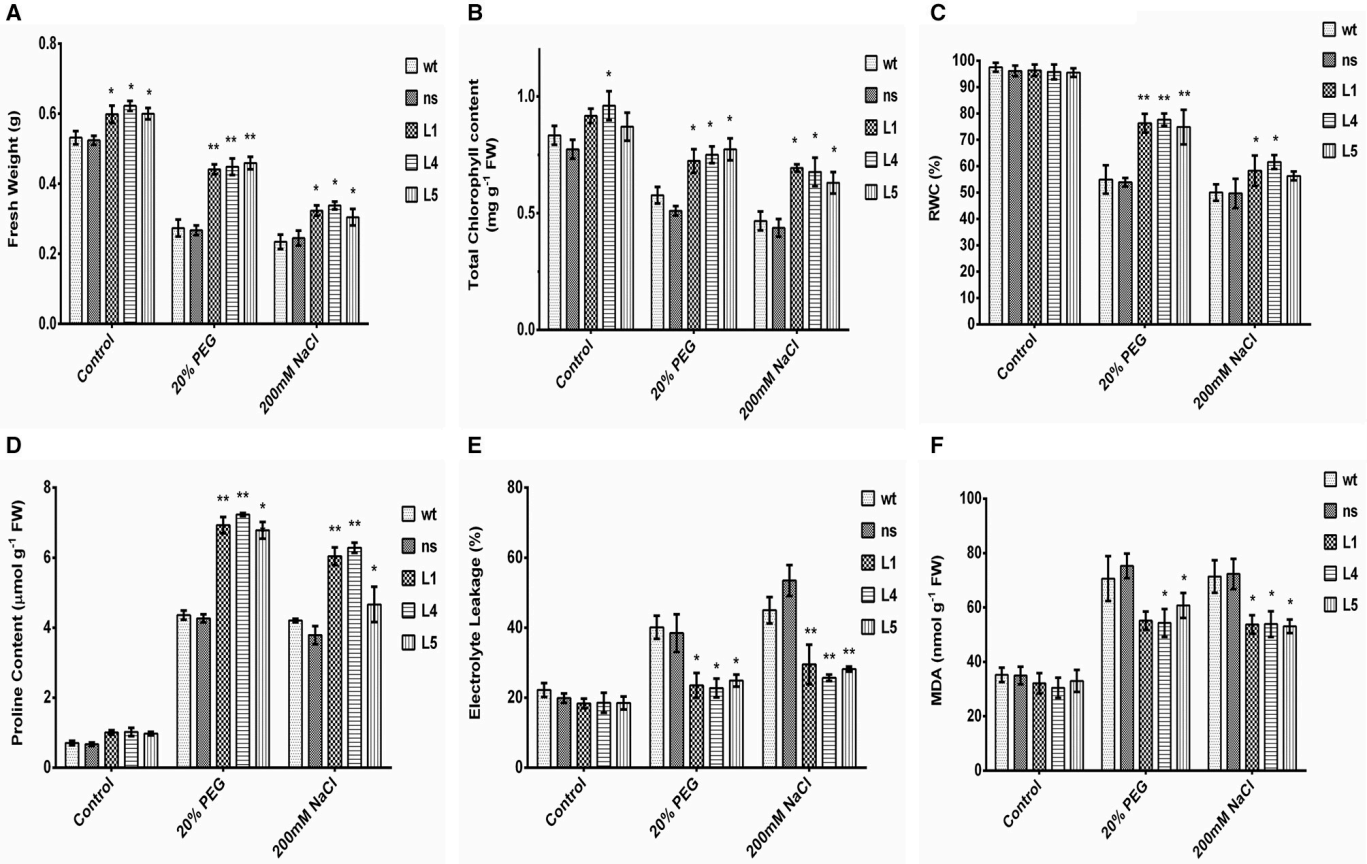
Various biochemical and physiological parameters of 2-week-old seedlings of *wt, ns*, and three *OsGS1;1/OsGS2* co-overexpressing transgenic rice lines (L1, L4, and L5) assessed after 2 days of osmotic (20% PEG) or salinity stress (200 mM NaCl) treatments as compared to untreated control conditions. **(A)** Fresh weight (FW) (in g). **(B)** Total chlorophyll content (in mg/g FW). **(C)** Relative water content (RWC) (in %). **(D)** Proline content (in μmol/g FW). **(E)** Electrolyte leakage (in %) **(F)** Malondialdehyde (MDA) content (in nmol/g FW). All data represented are means ± SD (*n* = 3). Asterisks above bars indicate significant differences from *wt* (*p-value ≤ 0.05 and **p-value ≤ 0.01).

To further study the physiological impact of *OsGS1;1/OsGS2* co-overexpression on drought and salinity tolerance in rice, 2-week-old transgenic seedlings and control seedlings were grown hydroponically and subjected to high osmotic stress (20% PEG for 4 days) or high salinity stress [200 mM NaCl (EC ~19 dS/m) for 4 days] followed by 4 days of recovery in untreated YS. Various physiological and biochemical parameters were assessed after 2 days of high osmotic and salinity stress treatments. The *OsGS1;1/OsGS2* co-overexpressing transgenic plants showed tolerant phenotypes and were able to recover better after high osmotic and salinity treatments, whereas the *wt* and *ns* control seedlings wilted and died (Figures 3D,E). Furthermore, the total fresh weight and chlorophyll content was significantly higher in the transgenic plants under both high salinity and osmotic stresses in comparison to the control. Transgenic lines had 61–67% higher fresh weight and 25–34% higher chlorophyll content compared to the corresponding control seedlings in the presence of 20% PEG induced osmotic stress (Figures 4A,B). Similarly, the transgenic seedlings maintained 29–44% higher fresh weight and 35–48% higher chlorophyll compared to the control seedlings under 200 mM NaCl induced salinity stress (Figures 4A,B). Interestingly, even under control conditions, the transgenic lines had around 12–17% higher fresh weight and 4–15% increased in chlorophyll content as compared to the corresponding non-transgenic control seedlings (Figures 4A,B).

Relative water content (RWC) is a quick method to determine the plant water status and gives an estimate of the cellular hydration levels after stress treatments (Barrs and Weatherley, 1962). The *OsGS1;1/OsGS2* co-overexpressing transgenic lines were able to maintain 74–78% RWC as compared to the corresponding control seedlings which maintained only 53–55% of RWC under 20% PEG induced high osmotic stress (Figure 4C). Whereas, under high salinity stress (200 mM NaCl), the transgenic seedlings maintained 56–61% of RWC compared to the corresponding control seedlings which had around 49–51% of RWC (Figure 4C). The higher RWC in transgenic seedlings under stressed conditions highlight the enhanced cellular hydration levels and physiological fitness compared to the corresponding non-transgenic control seedlings. Proline accumulation has been correlated with stress tolerant phenotype in many plants and acts as a compatible solute to protect the cellular machinery from stress induced damage (Hayat et al., 2012). In the present study, the relative proline content increased significantly (59–69%) in transgenic as compared to control seedlings in the presence of high osmotic stress (Figure 4D). Similarly, under high salinity stress, the transgenic lines showed increased proline content (59–65%) compared to *wt* control plants.

Electrolyte leakage provides an estimate of the cellular membrane stability during stress conditions (Bajji et al., 2002). The *OsGS1;1/OsGS2* co-overexpressing transgenic rice seedlings showed 34–43% lower rate of electrolyte leakage after treatment with either 20% PEG or 200 mM NaCl, in comparison to corresponding *wt* control seedlings exposed to the same stress conditions (Figure 4E). Also, the transgenic seedlings had 14–23% reduced MDA contents than *wt* control seedlings after high osmotic stress treatment and also 24–26% reduced MDA content than *wt* control seedlings after high salinity stress (Figure 4F). Malondialdehyde (MDA) is the product of decomposition of polyunsaturated fatty acids present in membranes as a result of free radical chain reactions and lipid peroxidation of biomembranes during stress conditions. The amount MDA content gives an estimate of the extent of lipid peroxidation and membrane injury that has occurred during adverse abiotic stresses. Overall, the assessment of various stress responsive physiological and biochemical parameters showed that *OsGS1;1/OsGS2* co-overexpressing transgenic rice seedlings had enhanced tolerance to osmotic and salinity stresses.

### *OsGS1;1/OsGS2* co-overexpressing rice transgenics showed enhanced tolerance to methyl viologen induced photo-oxidative stress

**Figure 5:**
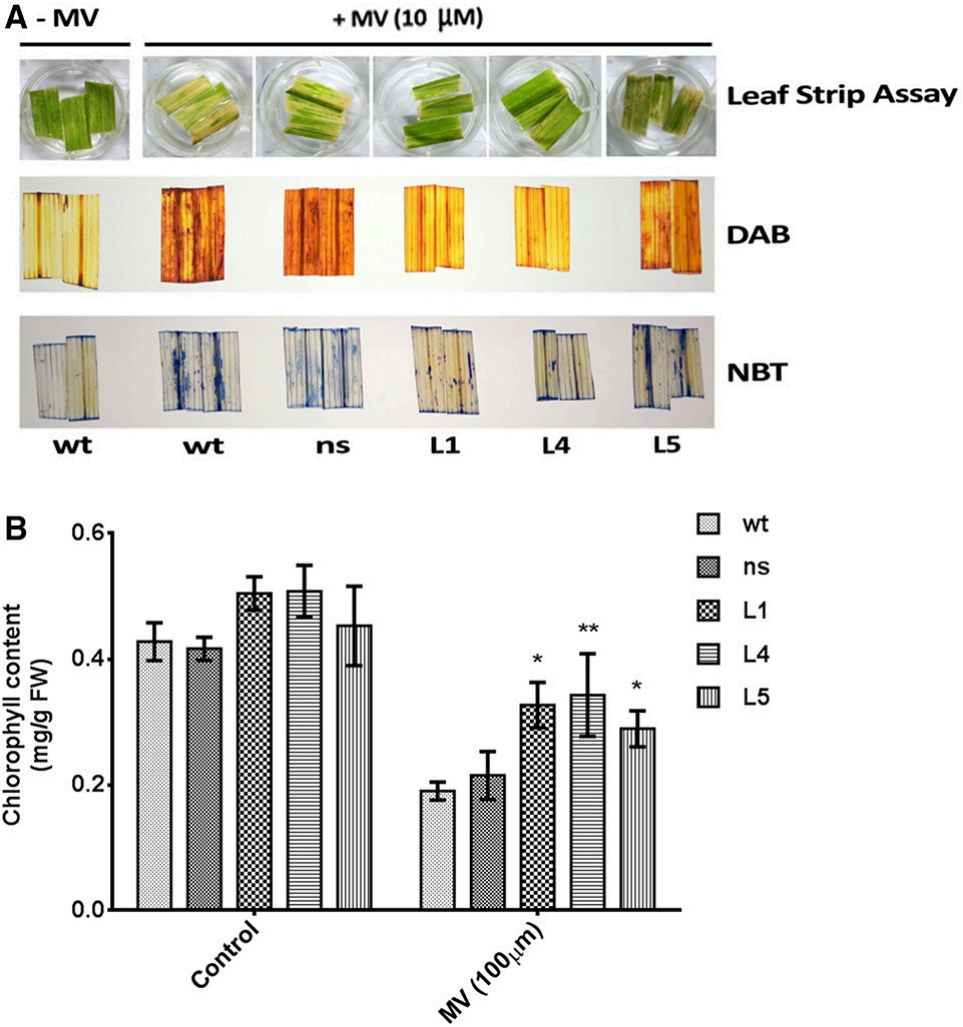
Assessment of tolerance of *OsGS1;1/OsGS2* co-overexpressing transgenic rice to methyl viologen (MV) induced photo-oxidative stress. (**A**; top panel) Leaf strips of *wt, ns*, and three T_2_ transgenic lines (L1, L4, and L5) after photo-oxidative stress treatment (incubation in 10 μM MV). Untreated (–MV) *wt* was used as a control (**A**; middle panel) Histochemical assessment of *in vivo* H_2_O_2_ formation following MV treatment by DAB staining. (**A**; bottom panel) *In vivo* generation of O${}_{2}^{-}$ in leaf strips after MV treatment as detected by NBT staining. **(B)** Total chlorophyll contents (in mg/g FW) after MV treatment as compared to untreated controls. Data represented are means ± SD (*n* = 3). Asterisks above bars indicate significant differences from *wt* (*p-value ≤ 0.05 and **p-value ≤ 0.01).

To assess the impact of photo-oxidative stress on the transgenic plants, uniform leaf strips harvested from the fully expanded leaves from *OsGS1;1/OsGS2* co-overexpressing rice seedlings and their corresponding *ns* and *wt* control rice seedlings were pre incubated with 10 μM methyl viologen (MV; paraquat) and exposed to sunlight. The leaf strips harvested from *wt* and *ns* control seedlings showed faster chlorosis than the transgenic seedlings (Figure 5A). To estimate the amount of *in vivo* ROS formation after treatment, the MV treated leaf strips were histochemically stained with DAB and NBT to estimate the amount of the ROS generated in response to the photo-oxidative treatment. H_2_O_2_ in the presence of peroxidases oxidizes DAB which leads to the production of a reddish brown precipitate. Similarly, the O${}_{2}^{-}$ free radical reacts with NBT to form a dark blue insoluble formazan compound (Kumar et al., 2014). The staining intensity of these tissues provides an estimate of *in vivo* H_2_O_2_ and O^2−−^ formation in response to MV or in response to any other stress. The transgenic lines showed significantly lower ROS production than *wt* and *ns* controls after MV treatment as evident by DAB and NBT staining (Figure 5A). The transgenic plants could also retain higher chlorophyll content due to protection from photo-oxidative damage through lesser ROS production after the MV treatment compared to *wt* and *ns* controls (Figure 5B). Thus, our preliminary results suggests that *OsGS1;1/OsGS2* co-overexpression in transgenic rice plants enhanced tolerance against MV induced photo-oxidative stress by modulating oxidative stress responses that mitigate excessive ROS formation.

### *OsGS1;1*/*OsGS2* co-overexpression improved agronomic performance under drought and salinity stresses at the reproductive stage

**Figure 6:**
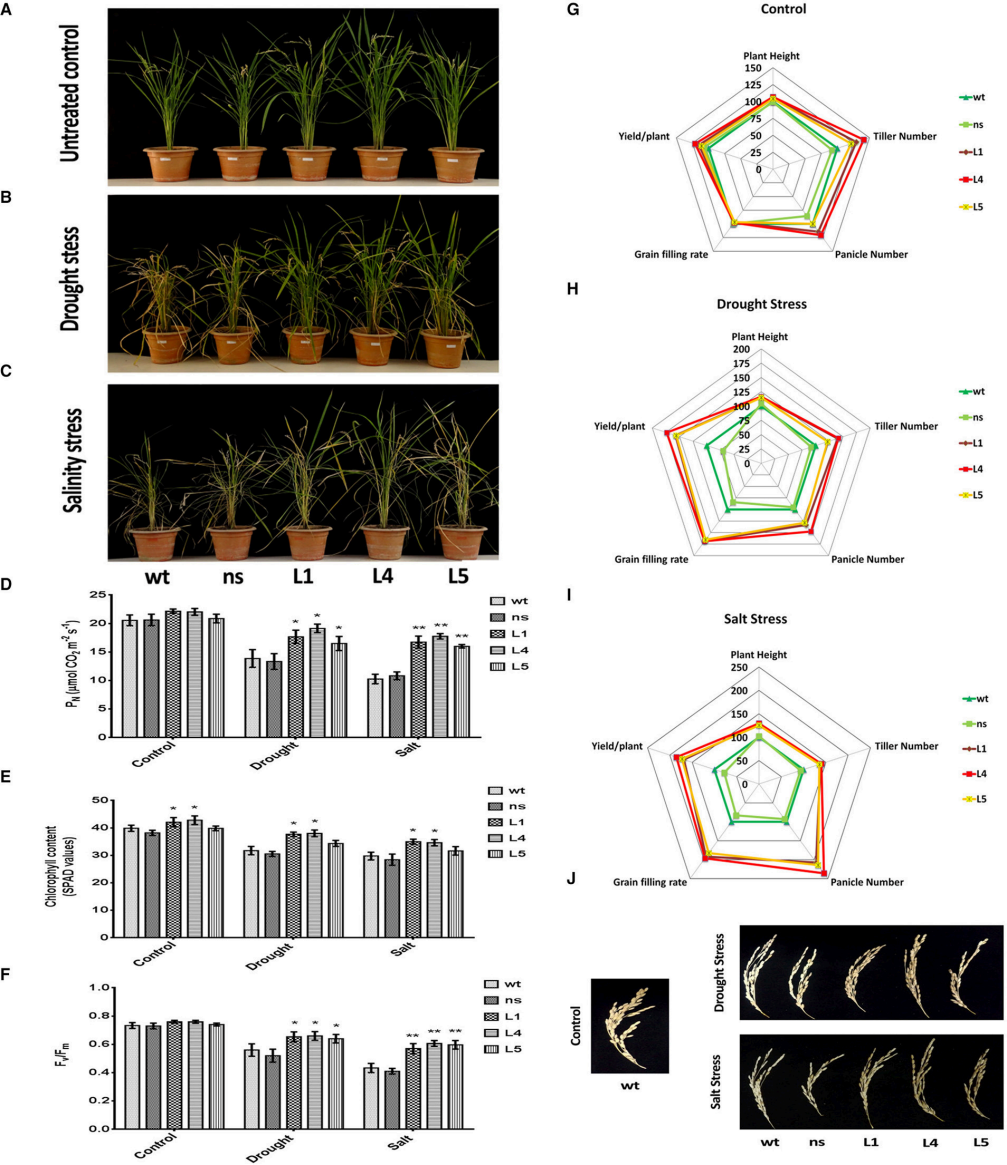
Agronomic and physiological performance of *OsGS1;1/OsGS2* co-overexpressing transgenic rice plants under abiotic stresses at reproductive stage. **(A)** Phenotypes of wild type (*wt*), null segregant (*ns*), and three transgenic lines (L1, L4, and L5) at reproductive stage under untreated control conditions. (B) Phenotypes after recovery for 15 days following drought stress treatment imposed by water withdrawal for 12 days post panicle initiation. **(C)** Phenotypes after recovery following moderate salinity stress imposed on ~2-month-old plants by irrigating pots every fortnight with water supplemented with 50 mM NaCl (EC~6 dS/m) until booting stage. Various physiological parameters such as **(D)** net photosynthetic rate (P_N_) (in μmol CO_2_/m^2^/s) **(E)** chlorophyll content (in SPAD values) and **(F)** chlorophyll fluorescence (F_v_/F_m_) assessed under control, drought and salinity stress conditions. All data represented are means ± SD (*n* = 3). Asterisks above bars indicate significant differences from *wt* (*p-value ≤ 0.05 and **p-value ≤ 0.01). Spider plots of agronomic traits of three independent T_2_ transgenic lines (L1, L4, and L5) and corresponding *ns* and *wt* controls under **(G)** untreated control **(H)** drought and, **(I)** salinity stress conditions respectively. Data plotted are percentages of mean values (*n* = 5). Mean values from *wt* plants were set at 100% as reference. **(J)** Grain filling phenotypes in *wt, ns*, and three transgenic lines (L1, L4, and L5) after recovery from drought and salinity stress as compared to untreated *wt* control.

We imposed terminal drought stress at the post panicle emergence stage by withdrawing irrigation for a period of 12 days to a set of *OsGS1;1/OsGS2* co-overexpressing transgenic and corresponding control rice plants until apparent differences in wilting and leaf rolling occurred. Following the drought treatment, the plants were recovered by regular irrigation. Another set of plants, which were regularly irrigated were kept as untreated control (Figure 6A). The transgenic plants were able to withstand the drought stress treatment and recovered vigorously post irrigation, whereas the *wt* and *ns* controls which had pronounced leaf rolling and wilting, recovered slowly (Figure 6B). The transgenic lines were able to maintain significantly (19–38%) higher net photosynthetic rates (P_N_) than the control lines after recovery from the drought stress (Figure 6D). The chlorophyll content as estimated by SPAD meter reading after drought treatment showed that the transgenic lines had 8–20% higher chlorophyll content than the control plants (Figure 6E). Chlorophyll fluorescence (Fv/Fm) gives an estimate of photodamage related effects of abiotic stress on the photosynthetic machinery. The chlorophyll fluorescence (Fv/Fm) ratios for the transgenic rice plants were significantly higher compared to both *wt* and *ns* controls under drought stress (Figure 6F). After drought stress recovery, transgenic rice lines showed significantly better agronomic performance with higher panicle number, grain filling rates and yield than *wt* and *ns* controls (Figure 6H). The transgenic lines had 28–48% higher panicle numbers than control plants and also showed significantly higher (64.6 ± 4.7%) grain filling rate compared to that controls (35.7 ± 2.8%) after recovery from drought stress (Figures 6H,J). The overall yield gain by the transgenic lines was 62.32 ± 9.65% higher than control plants after drought stress recovery (Figure 6H).

Similarly, we monitored the physiological fitness and agronomic performance of *OsGS1;1/OsGS2* co-overexpressing transgenic lines and the corresponding control plants under salinity stress by irrigating with 50 mM NaCl solution until the booting stage, followed by recovery. Another set of plants were irrigated with normal deionized water (untreated controls), grown to maturity and agronomic parameters assessed (Figure 6G). The transgenic lines showed improved growth parameters like significantly higher plant height, tiller number, panicle number, and yield than *wt* and *ns* controls under the salinity stress (Figure 6C). Under the salinity stress, the transgenic lines showed 25–29% higher plant height, 35–40% more tiller numbers, and 107–135% higher panicle number compared to *wt* and *ns* control plants (Figure 6I). Moreover, grain filling rates were significantly improved in transgenic lines, which had around 58.2 ± 4.5% as compared to 28 ± 10.6% in the controls (Figures 6I,J). The overall grain yield was 75.4 ± 8.7% higher in transgenic lines compared to *wt* and *ns* control plants under the salinity stress (Figure 6I). The transgenic lines were also able to maintain significantly (55–73%) higher net photosynthetic rates (P_N_) than the control lines after recovery from salinity stress (Figure 6D). The chlorophyll content of the transgenic lines was 6–17% higher in comparison to controls (Figure 6E). Also, the chlorophyll fluorescence, i.e., the Fv/Fm ratio was comparatively higher in transgenic plants as compared to both *wt* and *ns* control rice plants under salinity stress (Figure 6F). Our results show that the enhanced agronomic yield parameters in the *OsGS1;1/OsGS2* co-overexpressing transgenic rice plants correlated with the increased photosynthetic rates of transgenic lines in comparison to control plants, which suggests that the co-overexpression of *OsGS1;1/OsSG2* provided efficient photo-oxidative protection to the transgenic rice plants under reproductive stage drought and salinity stress.

### Simultaneous co-overexpression of *OsGS1;1/OsGS2* in rice conferred limited tolerance to PPT

**Figure 7:**
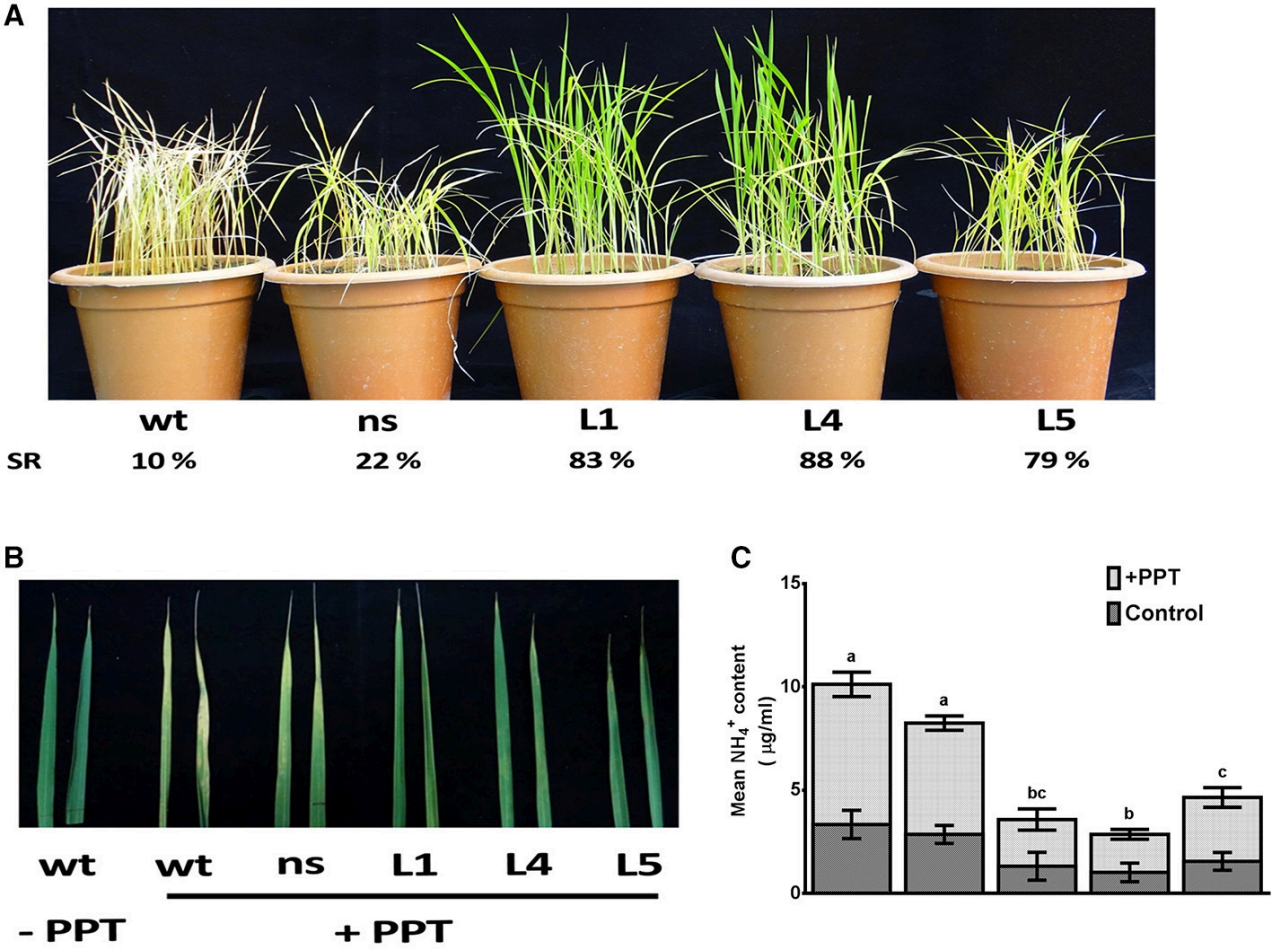
Assessment of tolerance of *OsGS1;1/OsGS2* co-overexpressing transgenic rice to phosphinothricin (PPT). **(A)** Phenotypes of *wt, ns*, and three T_2_ transgenic (L1, L4, and L5) rice seedlings after 0.5% (v/v) Basta herbicide (Glufosinate/PPT) spraying. Survival rates (SR) after spraying are indicated as percentages. **(B)** Mature leaves of *wt, ns*, and three T_2_ transgenic lines (L1, L4, and L5) painted with a solution of 0.5% Basta (v/v) (Bayer, 13.5% ai) supplemented with 0.01% Tween-20. **(C)** Mean NH${}_{4}^{+}$ liberation from leaves before and after PPT treatment. Data represented are the means ± SD (*n* = 3). Different letters above bars indicate significant difference among means of PPT treated group (Tukey-Kramer tests, *p*-value < 0.05).

The *OsGS1;1/OsGS2* co-overexpression in transgenic rice and control seedlings at four-six leaf stage were sprayed with 0.5, 1, and 2% (v/v) solution of Basta (Bayer, 13.5% ai). Transgenic lines had increased survival rates of 79–88% as compared to 10–22% of control plants after spraying with 0.5% (v/v) Basta solution (Figure 7A). The survival rate of line L5 was comparatively lower than the other lines, which could be as a result of the lower total GS enzymatic activity of this transgenic line. However, it was observed that the transgenic lines could not tolerate higher concentrations of 1% and 2% (v/v) Basta spray and all the transgenic lines along with the controls wilted by the 4th day after spraying (not shown). The mature leaves of both control and transgenic plants were also painted with a solution of 0.5% Basta supplemented with 0.01% Tween-20. After 5 days of treatment, the leaves were scored for visible injuries such as chlorosis, curling, bleaching and leaf burning on the painted areas; these symptoms were more prominent in *wt* and *ns* control seedlings than transgenic lines (Figure 7B). Thus, our results showed that the *OsGs1;1/OsGS2* co-overexpressing transgenic rice plants had limited tolerance to 0.5% (v/v) Basta (Glufosinate/phosphinothricin) herbicide.

The active ingredient of Basta (PPT/phosphinothricin) inhibits GS activity and prevents the re-assimilation of cytotoxic ammonium produced during various cellular metabolic processes including photorespiration. We monitored the accumulation of ammonium content in transgenic and control rice leaves before and after Basta (PPT) spray. We estimated 3.1 ± 0.3 μg/mL free ammonium in control rice leaf samples, whereas, 1.2 ± 0.2 μg/mL in *OsGS1;1/OsGS2* co-overexpressing transgenic rice leaf samples before spraying the 0.5% Basta solution (Figure 7C). However, after Basta spraying, the ammonium content significantly accumulated to 9.1 ± 3.6 μg/mL in control rice leaf samples, whereas, only a marginal increase (3.6 ± 0.8 μg/mL) was observed in *OsGS1;1/OsGS2* co-overexpressing transgenic rice (Figure 7C).

## Discussion

### Abiotic stress tolerance in GS overexpressing plants: possible mechanistic routes to enhanced tolerance and yield

Our results demonstrate that concurrently overexpressing both the cytosolic *OsGS1;1* and chloroplastic *OsGS2* isoforms in transgenic rice, enhanced its tolerance against drought and salinity stresses during seedling and reproductive stages, and against MV induced photo-oxidative stress. The transgenic plants showed higher chlorophyll fluorescence (Fv/Fm) under drought and salinity stress as compared to the corresponding control rice plants (Figure 6F), suggesting that the transgenic lines had enhanced protection of the photosynthetic machinery, which thereby led to improved recovery post stress. These results corroborate several other previous studies, which highlight the role of GS overexpression in conferring photoprotection of the photosynthetic machinery during various abiotic stresses. For instance, transgenic rice overexpressing the chloroplastic *OsGS2* gene was shown to have improved tolerance to salinity and chilling stress due to increased photorespiratory capacities (Hoshida et al., 2000), while the ectopic overexpression of a pine cytoplasmic GS1 gene in transgenic poplar conferred improved tolerance to drought stress (El-Khatib et al., 2004). Similarly, Kozaki and Takeba (1996) reported that transgenic tobacco plants over-expressing the chloroplastic GS2 displayed enhanced tolerance to high light intensity. They determined that the overexpression of GS2 led to improved re-assimilation of photorespiratory ammonia, resulting in better protection of photosynthesis by reducing the damage due to photo-oxidation on the photosynthetic apparatus. Furthermore, several lines of evidence show that the rate-limiting step in photorespiration is likely to be the re-assimilation of NH${}_{4}^{+}$ catalyzed by the chloroplastic GS2 (Wallsgrove et al., 1987; Häusler et al., 1994; Kozaki and Takeba, 1996). For instance, mutant barley plants lacking the chloroplastic GS2 isoform were found to have severely reduced photorespiratory capacities (Wallsgrove et al., 1987). Photorespiration is thought to function as a potential route for the dissipation of excess light energy or reducing power (NADPH) generated during various abiotic stresses (Osmond and Grace, 1995; Willekens et al., 1997; Wingler et al., 2000; Voss et al., 2013). During various abiotic stress conditions, such as drought, salinity, or high light, the reducing equivalents (NADPH) generated through photosynthetic light reactions often surpasses the demand of the Calvin Benson Bassham cycle. The excess energy thus generated is dissipated as heat or the electrons are transferred from various complexes in the electron transport chain to other acceptor molecules such as O_2_, which would thereby result in the production of excessive reactive oxygen species (ROS) (Peterhansel et al., 2010). Thus, under such stress conditions, photorespiration can apparently act as an electron sink by consuming the excess electrons or reducing equivalents through the re-assimilation of photorespiratory NH${}_{4}^{+}$ by chloroplastic GS2 (Wingler et al., 2000; Peterhansel et al., 2010). The overexpression of GS2 would thus enhance photoprotection since excess electrons generated by photo-oxidation during stresses would be diverted from O_2_ to the photorespiratory cycle which would lead to the reduction in ROS generation, and thereby enhance tolerance to abiotic stresses (Hoshida et al., 2000). Our findings reveal that under photo-oxidative stress induced by methyl viologen, *OsGS1;1/OsGS2* co-overexpressing transgenics had significantly enhanced tolerance and reduced ROS production as evidenced by leaf strip assays and DAB, NBT staining for H_2_O_2_ and O${}_{2}^{-}$ free radicals. Moreover, GS overexpression may also lead to reduction of ROS levels by modulating the oxidative stress response enzymes involved in antioxidant formation. Lee et al. (2013) previously reported that *OsGS1;1* overexpressing transgenic rice had enhanced tolerance to cadmium induced oxidative stress through the modulation of oxidative stress responses. In addition, transgenic poplar plants overexpressing GS were shown to differentially regulate the expression of genes involved in mitigation against ROS, such as copper-dependent super oxide dismutases, thereby significantly improving their tolerance against drought stress (Molina-Rueda et al., 2013; Molina-Rueda and Kirby, 2015).

Also, GS overexpression is likely to increase the flux through the GS-GOGAT cycle, which would thereby increase net glutamate and glutamine amino acid levels. Several GS overexpression studies in plants have reported associated increases in total free amino acid levels especially of glutamate and glutamine (Migge et al., 2000; Fuentes et al., 2001). The amino acid glutamate is required for the synthesis of osmoprotectants such as proline, polyamines, as well as glutathione (GSH) (a tripeptide containing glutamate, cysteine, and glycine), a major intracellular antioxidant. A higher free glutamate amino acid level is essential for enhanced GSH levels which would in turn help in mitigation of excessive ROS production during stress conditions. Moreover, increased photorespiratory activity of GS overexpressing plants would also result in the synthesis of sufficient amounts of glycine, which has enhanced demand during GSH synthesis under abiotic stress conditions (Noctor et al., 1997). In addition, higher glutamine levels have also been shown to induce the transcription of stress responsive transcription factors such as DREB1A, IRO2, and NAC5 involved in various abiotic stresses (Kan et al., 2015), which is another possible mechanism by which GS overexpression leads to enhanced tolerance to abiotic stresses.

Membrane lipid peroxidation due to enhanced ROS activity and subsequent membrane leakage is common during various abiotic stresses and causes severe damage to plant cells leading eventually to cell death. However, the *OsGS1;1* and *OsGS2* co-overexpressing rice transgenic lines in our study showed significantly lower MDA content and lesser electrolyte leakage under drought and salinity stress compared to control rice plants under the same stress condition (Figures 4E,F). The observed membrane stability and lower lipid peroxidation in transgenic rice lines is likely to be due to lower ROS generation under drought and salinity stress. Our result corroborates reports that transgenic rice lines constitutively overexpressing the cytosolic *OsGS1;1* gene had significantly lower MDA levels under cadmium stress (which is considered to be an oxidative stress enhancing factor), thus suggesting that GS overexpression modulates oxidative stress responses (Lee et al., 2013).

The *OsGS1;1/OSGS2* co-overexpressing rice transgenics in our study, also accumulated significantly higher proline contents than controls under salinity and drought stresses (Figure 4D). The accumulation of proline and polyamines is a common response to various abiotic stresses (Vinocur and Altman, 2005). They act as osmolytes and help in protecting cellular membranes and proteins under various stresses (Yoshiba et al., 1997; Sengupta et al., 2016). It has been demonstrated that GS plays a key role in regulating proline production in the phloem and that higher GS activity is essential to synthesizing proline under water stress (Larher et al., 1998; Brugiere et al., 1999). Mutants of Lotus plants, deficient in chloroplastic GS2 had significantly lower proline contents than wild type plants during drought stress, which led to their compromised recovery following re-watering (Díaz et al., 2010). Moreover, poplar cells treated with a GS enzyme inhibitor (methionine sulphoximine) caused a reduction of the polyamine content which suggested that polyamine levels in plants are also primarily regulated by GS (Bhatnagar et al., 2001). Thus, increased accumulation of such osmoprotectants may be another possible mechanism of tolerance in GS overexpressing plants to abiotic stress.

The *OsGS1;1*/*OsGS2* co-overexpressing rice lines were also able to withstand terminal drought stress and salinity stress and recovered vigorously with significant increases in tiller number, panicle number, better grain filling, and showed overall yield improvement in comparison to corresponding control plants (Figures 6A–I). As we observed better grain filling rates in transgenic plants over controls under abiotic stresses, we postulate that sufficient GS1;1 activity in the shoots combined with enhanced GS2 activity in the chloroplast in *OsGS1;1/OsGS2* co-overexpressing transgenic rice plants, may lead to improved N re-assimilation into sink tissues and better photoprotection of photosynthetic machinery thereby leading to better recovery post abiotic stresses and consequently to an increase in grain filling and yield under stresses. Furthermore, under abiotic stress conditions, increased cellular processes like proteolysis can result in high intracellular ammonia, causing toxicity if not removed efficiently (Lutts et al., 1999). Enhanced GS activity would potentially alleviate this toxicity and at the same time improve N recycling efficiency, maintain photosynthetic enzymes and thereby lead to better growth and yield (Fuentes et al., 2001). The maintenance of photosynthesis at higher rates post recovery from abiotic stresses as observed in the *OsGS1;1*/*OsGS2* co-overexpressing transgenic rice plants in comparison to stressed control plants also adds credence to this view (Figure 6D). Several GS overexpression studies have reported similar results in various transgenic plants (Migge et al., 2000; Fuentes et al., 2001; Habash et al., 2001; Oliveira et al., 2002; Martin et al., 2006; Cañas et al., 2010; Brauer et al., 2011). In addition, several quantitative trait loci (QTL) previously mapped and implicated in crop yield and growth components were shown to co-localize to the GS loci in maize (Hirel et al., 2001, 2007; Gallais and Hirel, 2004), rice (Obara et al., 2001, 2004; Yamaya et al., 2002), wheat (Habash et al., 2007; Bernard et al., 2008), and barley (See et al., 2002). Besides, the overexpression of the *OsGS1;2* isoform in rice was shown to increase spikelet number and percentage grain filling in comparison to azygous controls (Brauer et al., 2011). Thus, the enhanced GS activity in transgenic rice plants presumptively leads to improved photoprotection of the photosynthetic machinery, thereby leading to better recovery post abiotic stresses and consequently to an increase in grain filling and yield. Efficient partitioning and remobilization of N resources under stress conditions is likely to be enhanced by *OsGS1;1*/*OsGS2* co-overexpression which could also be the reason behind the enhanced agronomic performance of the transgenics in comparison to control plants.

In contrast, Cai et al. (2009) reported the transgenic overexpression of cytosolic *OsGS1;1* or *OsGS1;2* in rice showed very poor plant growth and reduced yield and had no significant tolerance to any abiotic stresses. This phenotype was seen to be due to imbalances in the carbon-nitrogen (C-N) metabolic status (Bao et al., 2014). It is important to note that the C-N metabolisms in the plant system are tightly interlinked and work in a concerted and regulated manner, evident from the fact that GS metabolism needs energy in the form of ATP and reduced ferredoxin or NADH, and also requires C-skeletons in the form of 2-oxoglutarate (2-OG), which are provided by C metabolism (Hodges, 2002). The GS-GOGAT cycle thus is believed to act as a linker between C and N metabolic cycles. It is very likely that GS overexpression leading to improvement of growth, yield and or abiotic stress tolerance would only be feasible in plants with higher GOGAT activity and/or enhanced supply of energy (ATP and NADH) and 2-oxoglutarate from C metabolism. The involvement of a highly coordinated carbon-nitrogen (C-N) metabolic system along with the intricate regulation of GS at various levels is often associated with the inconsistent GS overexpression studies under various plant genetic backgrounds (for a complete review see Thomsen et al., 2014).

### Overexpression of multiple GS isoforms for glufosinate herbicide tolerance: advantages and limitations

We observed that *OsGS1;1/OsGS2* co-overexpressing rice plants could tolerate Glufosinate herbicide (PPT) application to a limited extent (0.5% v/v Basta spray and painting) (Figure 7A). The lower accumulation of ammonia after PPT treatment in the transgenics as compared to *wt* showed that the overexpression of GS isoforms was able to partially detoxify the excess ammonia produced by PPT application to a limited extent. Several overexpression studies of GS in crops have been reported to show tolerance to the herbicide Glufosinate (PPT) which is a potent inhibitor of the GS enzyme. In rice, transgenic plants overexpressing the *OsGS1;2* gene under a *CaMV* 35S promoter showed resistance to 10 mg/L of Basta *in vitro* and 0.5% (v/v) solution of Basta applied as a foliar spray. However, it was seen that the overexpression of the *OsGS1;1* isoform alone did not result in Basta tolerance (Cai et al., 2009). Sun et al. (2005) reported that simultaneous overexpression of pea *GS1;1* and *GS2* in rice plants conferred resistance to 0.3% Basta solution painted on leaves. Similar results were seen in wheat plants simultaneously overexpressing both cytosolic and chloroplastic pea GS isoforms which tolerated upto 0.3% (v/v) Basta when painted on leaves (Huang et al., 2005). Also, transgenic poplar overexpressing a cytosolic pine GS showed considerable tolerance to a foliar application of PPT with enhanced growth in transgenic over controls (Pascual et al., 2008). Commercially available transgenic herbicide resistant crops have till date utilized the *bar* (bialaphos resistance) or *pat* (phosphinothricin acetyl transferase) derived from *Streptomyces* species for conferring resistance to the Glufosinate herbicide. For instance, Bayer's Liberty link Glufosinate resistant transgenic crops are one of the most successfully commercialized transgenics currently in use in modern agriculture. However, given the increasing bio-safety concerns dissuading the use of bacterial genes in food crops and the general public resentment against them, an alternative strategy for Glufosinate resistant herbicide crops would be the overexpression of the target gene (GS) or introducing mutations in GS for conferring tolerance against Glufosinate. But, when compared to resistance levels in plants obtained using the PPT detoxifying *bar* or *pat* genes the overexpression of GS as a strategy for developing Glufosinate resistant crops has shown unsuitably low resistance levels for commercial viability. One of the limitations facing this is the presence of multiple GS isozymes in different crop species (Donn and Köcher, 2002). As seen in our studies, gene stacking of multiple GS isoforms using the Multi-Round Gateway technology may help in overcoming this problem. Besides, several mutations in the GS enzyme have been seen to confer improved resistance to Glufosinate (Supplementary Table 2). For instance, recently a DNA shuffling of the *OsGS1;1* gene of rice under selective pressure of high concentrations of PPT, identified an arginine at 295 to lysine mutation (R295K) as responsible for conferring PPT resistance. Further complementation studies of this mutation in a GS mutant *Saccharomyces cerevisiae* and transgenic overexpression of the *OsGS1*;*1* R295K mutant gene in *Arabidopsis* confirmed its ability to confer high levels of tolerance to PPT (Tian et al., 2015). Therefore, multiple-gene stacking of such mutant GS isoforms may help in further developing the commercial viability of this strategy. However, due to the presence of multiple gene loci encoding for GS isozyme in plant species, with non-overlapping and non-redundant physiological roles in plant development, the introduction of targeted mutations to confer Glufosinate resistance in all the isoforms is a daunting task.

## Conclusion and future strategies

**Figure 8:**
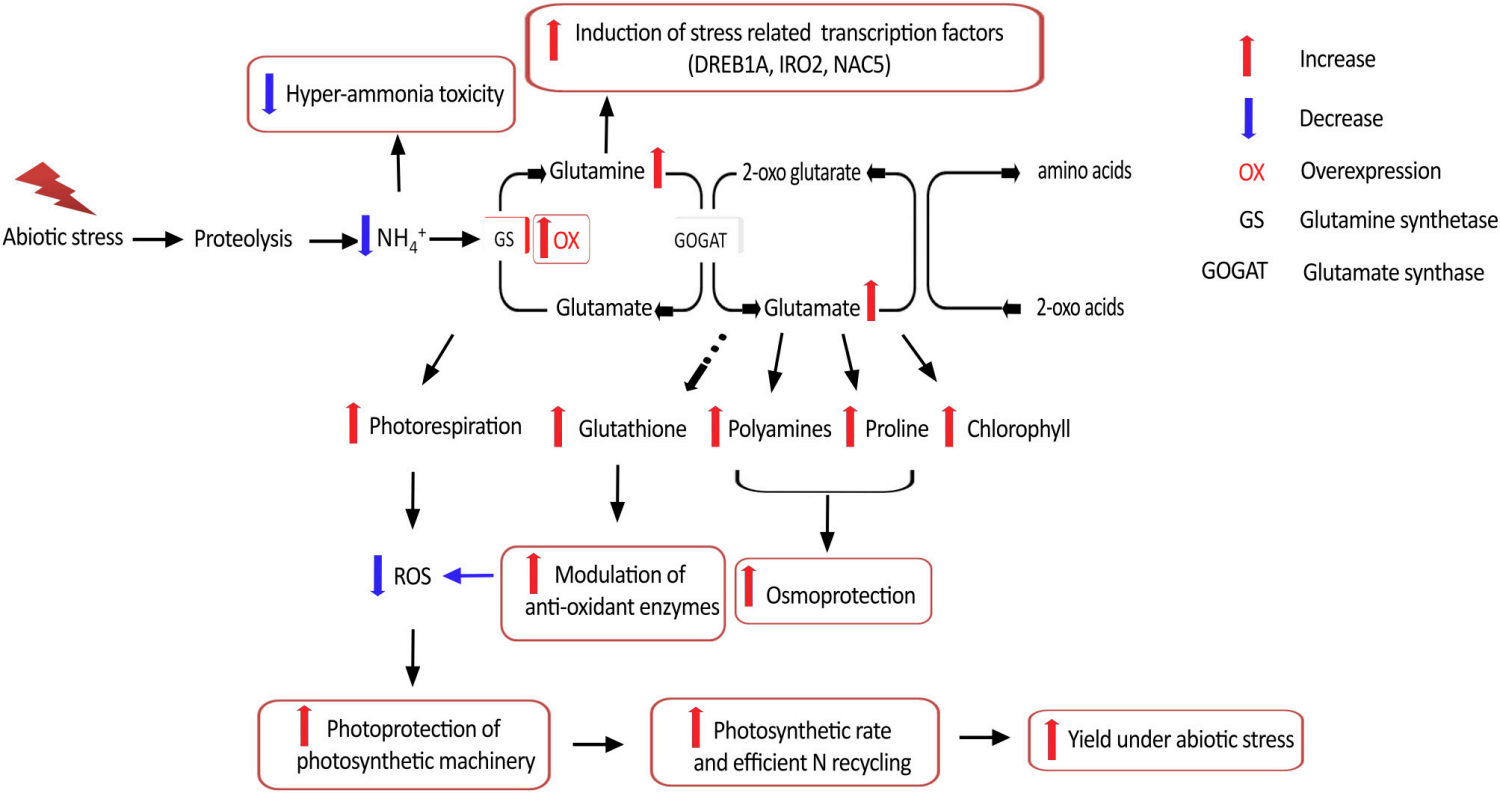
Putative mechanistic roles of GS in abiotic stress tolerance. Overexpression of GS alleviates hyper-ammonia toxicity caused due to proteolysis associated with various abiotic stresses. Since GS is considered the rate limiting step for photorespiration, GS overexpression can increase photorespiratory capacities and thereby ensuring photoprotection of the photosynthetic machinery via reduced reactive oxygen species (ROS) generation. GS overexpression has been reported to enhance production of the amino acids glutamine and glutamate, which is necessary for production of proline and polyamines which provide osmoprotection, as well as the anti-oxidant glutathione which can modulate anti-oxidant enzyme responses and thereby alleviate oxidative stress. Higher glutamine content is reported to induce expression of stress related transcription factors. Overexpression of GS is also reported to increase photosynthetic rates and is likely to improve N recycling efficiency, thereby leading to better yield under abiotic stress.

Taken together, our results suggest that *OsGS1;1/OsGS2* co-overexpression in rice conferred enhanced physiological tolerance and increased agronomic performance under abiotic stresses, apparently acting through multiple mechanistic routes (Figure 8). In addition, the co-overexpression of *OsGS1;1* and *OsGS2* in rice also conferred limited tolerance to the herbicide Glufosinate. However, a more comprehensive understanding of the regulation of GS and the functions of various isoforms in abiotic stresses would be needed before consistent results can be obtained across species and varieties. Moreover, since chloroplastic GS2 is itself prone to oxidative degradation by ROS under higher levels of stress (Palatnik et al., 1999; Ishida et al., 2002); the overexpression of a mutant GS resistant to inactivation under oxidative conditions might help in increasing abiotic stress tolerance of crop plants. Future overexpression strategies using GS for crop improvement will also have to take into consideration the complex regulation of GS and its intimate interaction with the C-N metabolic pathway to overcome potential metabolic bottlenecks. The use of more refined strategies, such as gene stacking in combination with developmental-stage and/or tissue specific expression, is expected to provide improved and consistent results (Thomsen et al., 2014). However, the prospect of transgenically manipulating GS to enhance yield and NUE under abiotic stress conditions, and provide field level herbicide resistance is lucrative enough to continue such directed efforts to tap the full potential of this unique enzyme in crop improvement for sustainable agriculture.

## Author contributions

MR and DJ conceived the project and all co-authors were involved in planning the experiments. DJ, DF, BB, BR, RY, and MM conducted the experiments and collected the data. DJ, JS, VP, VA, and VS performed data analysis and drafting of the manuscript. DJ, VA, and MR revised the final manuscript.

### Conflict of interest statement

The authors declare that the research was conducted in the absence of any commercial or financial relationships that could be construed as a potential conflict of interest.

## Abbreviations

Ai: Active ingredient
BCIP: 5-Bromo-4-chloro-3-indolyl phosphate
DAB, 3: 3-Diaminobenzidine
DIG: Digoxigenin
EC: Electrical conductivity
GS: Glutamine synthetase
MDA: Malondialdehyde
MW: Molecular Weight
NBT: Nitroblue tetrazolium
NUE: Nitrogen use efficiency
PAGE: Polyacrylamide gel electrophoresis
PBS: Phosphate buffered saline
PEG: Poly Ethylene Glycol
PPT: Phosphinothricin
QTL: Quantitative trait loci
ROS: Reactive oxygen species
RWC: Relative water content
SDS: Sodium dodecyl sulfate
WUE: Water use efficiency
YS: Yoshida hydroponics nutrient solution.
