# Supplementary material for: Concurrent Overexpression of OsGS1;1 and OsGS2 Genes in Transgenic Rice (Oryza sativa L.): Impact on Tolerance to Abiotic Stresses
Source: Front Plant Sci. 2018 Jun 21;9:786. doi: 10.3389/fpls.2018.00786 (PMC6021690; doi:10.3389/fpls.2018.00786)
Supplement: Supplementary file 1 [file Table_1.docx]

Supplementary Material

**Concurrent overexpression of *OsGS1;1* and *OsGS2* genes in transgenic rice (*Oryza sativa* L.): Impact on tolerance to abiotic stresses**

**Donald James^1^, Bhabesh Borphukan^1^, Dhirendra Fartyal^1&2^, Babu Ram^1&2^, Jitender Singh ^1&3^, Mrinalini Manna^1^, Vijay Sheri^1^, Panditi Varakumar^1^, Renu Yadav^1^, V. Mohan M. Achary^1^, Mallireddy K. Reddy^1*^**

* **Correspondence**: Mallireddy K. Reddy: [reddy@icgeb.res.in](mailto:reddy@icgeb.res.in)

**Supplementary Table 1**

**List of Primers**

| **OsGS1;1**  **cloning**  **primer** | Forward primer (Nco1)- 5’-GCGCCATGGCTTCTCTCACCGATCTCG-3’  Reverse primer (Not1)- 5’-TTAGCGGCCGCTCAGGGCTTCCAGATGAT-3’ |
| --- | --- |
| **OsGS2**  **cloning**  **primer** | Forward primer (Spe1)- 5’-GAGACTAGTATGGCGCAGGCGGTGGTG-3’  Reverse primer (Not1)- 5’-AATGCGGCCGCTCATACCTTCAGGGCCAACTTCTT-3’ |
| ***hpt***  **screening primer** | Forward primer- 5’- ATGAAAAAGCCTGAACTCACC -3’  Reverse primer -5’- CTATTTCTTTGCCCTCGGAC -3’ |
| **OsGS1;1:Act2T**  **screening**  **primer** | Forward primer- 5’- GCGCCATGGCTTCTCTCACCGATCTCG -3’  Reverse primer -5’- TTCTCATGAAAGATACAGCTAGGCCA -3’ |
| **OsGS2:Act1T**  **screening**  **primer** | Forward primer- 5’- GAGACTAGTATGGCGCAGGCGGTGGTG -3’  Reverse primer -5’- CTCCTCTTGGCTTAGCATTCTTGGGTCCGA -3’ |
| **OsGS1;1**  **RT-PCR primer** | Forward primer- 5’- TGTGGTATCGGTGCTGACAAG-3’  Reverse primer -5’- AACTCCCACTGTCCTGGCAT -3’ |
| **OsGS2**  **RT-PCR primer** | Forward primer- 5’- AGTGTCGGTATTGAAGCTGGAG-3’  Reverse primer -5’- ACGCATACTCTTGGTGCTGTAG -3’ |
| **eEF-1α**  **RT-PCR primer** | Forward primer- 5’- TTCCCTTTGTTCCCATCTCTGG-3’  Reverse primer -5’- TGATCTGGTCAAGAGCCTCAAG -3’ |

**Supplementary Table 2**

**Possible mutations of GS identified which confers resistance to PPT**

| **Mutation (Residue positions)** | **Reference** |
| --- | --- |
| 207 is Gly (X207G)  245 is anything other than Gly (X245≠G)  Gly 245 can preferentially be Ser/Cys/Arg  (G245S; G245C; G245R)  Arg 332 to Lys (R332K) | Goodman et al. 1990  (US Patent US4975374 A) |
| Glu 297 to Ala  (G297A)  (known as the glutamate loop) | Clemente and Márquez 1999 |
| Glu 304 to Ala/Asp (Glu loop)  (E304A; E304D)  Ala 305 Glu (also a part of Glu loop )  (A305E) | Wray and Fisher 2010 |
| His 249 Tyr (H249Y) | Pornprom et al. 2009 |
| Arg 295 to Lys (R295K) | Tian et al 2015 |

**Supplementary Figure S1**

**

**

Relative expression levels of *OsGS1;1* and *OsGS2* transcripts in *wt* and three transgenic rice lines (L1, L4 and L5) as quantified from densitometric analysis of band intensities of semi-quantitative RT-PCR gels using Image J.

**Supplementary Figure S2**





Relative protein contents of OsGS1;1 and OsGS2 in *wt* and three transgenic rice lines (L1, L4 and L5) as quantified from densitometric analysis of band intensities of immunoblots using Image J.

**Supplementary Figure S3**


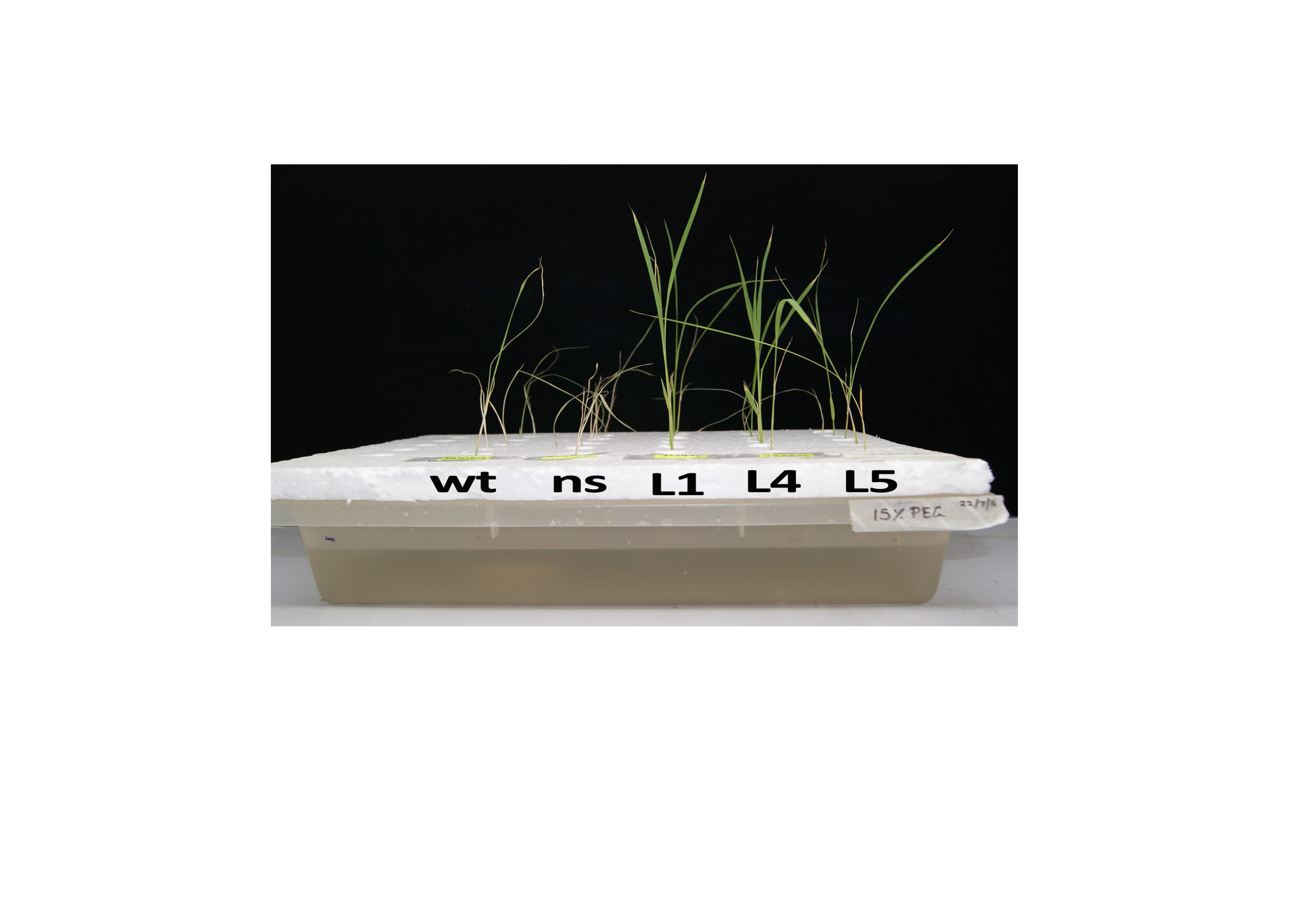


Phenotype of seedlings of *wt, ns* and three transgenic rice lines (L1, L4 and L5) 12 days after being grown hydroponically in Yoshida solution supplemented with 15% PEG.
